# Employing Ray-Tracing and Least-Squares Support Vector Machines for Localisation †

**DOI:** 10.3390/s18114059

**Published:** 2018-11-20

**Authors:** Benny Chitambira, Simon Armour, Stephen Wales, Mark Beach

**Affiliations:** 1 Communication Systems & Networks Group, University of Bristol, Bristol BS8 1UB, UK; simon.armour@bristol.ac.uk (S.A.); m.a.beach@bristol.ac.uk (M.B.); 2 Roke Manor Research, Romsey, Hampshire SO51 0ZN, UK; stephen.wales@roke.co.uk

**Keywords:** localisation, positioning, ray-tracing, support vector machine

## Abstract

This article evaluates the use of least-squares support vector machines, with ray-traced data, to solve the problem of localisation in multipath environments. The schemes discussed concern 2-D localisation, but could easily be extended to 3-D. It does not require NLOS identification and mitigation, hence, it can be applied in any environment. Some background details and a detailed experimental setup is provided. Comparisons with schemes that require NLOS identification and mitigation, from earlier work, are also presented. The results demonstrate that the direct localisation scheme using least-squares support vector machine (the Direct method) achieves superior outage to TDOA and TOA/AOA for NLOS environments. TDOA has better outage in LOS environments. TOA/AOA performs better for an accepted outage probability of 20 percent or greater but as the outage probability lowers, the Direct method becomes better.

## 1. Introduction

Localisation in multipath environments has been a challenge for mobile wireless systems. Global Navigation Satellite Systems (GNSS) like GPS have been the default go-to technologies for localisation and navigation, but these have also performed poorly in urban areas because of multipath propagation and blockage of the satellite in urban canyons. GNSS systems are not able to provide indoor localisation and navigation. Multi-GNSS systems improve location accuracy, but still have been reported to have achieved a best-case 2-D accuracy of 40m, in a worst-case urban canyon environment [1]. These issues have instigated research into mobile wireless localisation.

Positioning systems that make use of mobile radio systems determine the location by first determining the range (distance from the base-station (BS)) of the mobile station (MS) or user equipment (UE). Time delay measurements or the power level of the received signal are used in trilateration and Angle-of-Arrival (AOA) measurements are used in triangulation. For triangulation, the challenge is to determine the true line-of-sight (LOS) angle of arrival at the BS, which is a difficult task in urban multipath environments, when all signals arriving at the BS are in non-line-of-sight (NLOS). In trilateration, the time delay measurements generally consist of a positive bias due to multipath. Both of the above cases give rise to the need for NLOS identification and mitigation.

The use of Least-Square Support Vector Machines (LSSVMs) to address the challenges of localisation in NLOS environments is becoming popular. Already LSSVMs have been shown to be effective for NLOS identification and mitigation in urban environments [2]. In this article, we show a direct scheme, that provides for localisation of the mobile station, without needing to go through NLOS identification and mitigation processes [3]. We also compare the performance of both approaches in different environments. Ray tracing data for the greater city of Bristol is used in the study. We use NLOS identification and mitigation, and the localisation algorithms discussed in [2], to compare with the Direct method. Detailed discussion on other NLOS identification and mitigation techniques can be found in [4].

Related approaches like fingerprinting [5] involve matching the received signal quantities, commonly the received signal strength, to the values that are pre-recorded in the fingerprinting database, for any particular environment. A fingerprinting database is built by taking or collecting measurements per grid, of the area of interest. Positioning accuracy in this scheme, therefore, depends on the grid size used. Fingerprinting requires cell matching before correlation with grids around that cell, whereas the LSSVM method discussed here, handles BS matching and location estimation within the same framework. Also, fingerprinting employs either probabilistic algorithms, such as maximum likelihood, to estimate the position or deterministic algorithms that calculate the similarity between the UE measurement and the database grid-based measurements. Because of the challenges and limitations of fingerprinting, it is commonly considered as an augmenting scheme to other approaches to improve accuracy. Ray-tracing has also been demonstrated to be an effective approach for localisation, including when used together with finger-printing [6,7,8].

## 2. Experimental Setup and Methodology

### 2.1. Ray Tracing

**Figure 1:**
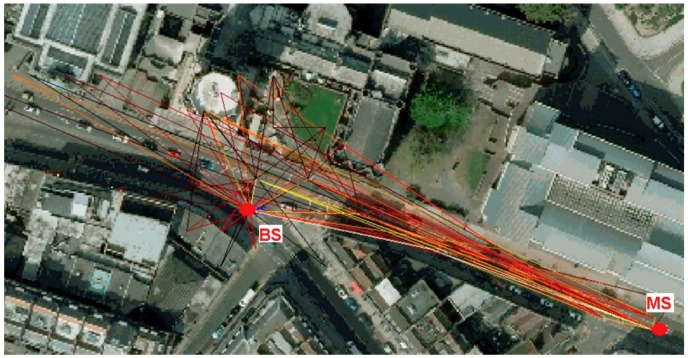
Multipath rays for a BS-MS point-to-point link.

Ray-tracing software that was developed at the University of Bristol, is used in this study. It is based on a validated, realistic 3D ray-traced channel model as used in [9,10]. The same ray-tracing model was used to generate most of the statistics that are now specified in the 3D extension of the 3GPP/ITU channel model [11]. The ray tracing tool incorporates a real-world environmental database of the City of Bristol (UK), which is a 3D Laser Illuminated Detection & Ranging (LIDAR) database of the city. Figure 1 shows, as an example, a point-to-point BS-MS link with all the determined rays (multipath rays) between the BS and the MS.

Ray-tracing is conducted at a carrier frequency of 3.51 GHz to match the carrier frequency used on the Bristol massive MIMO testbed [12]. The transmit power is set to 32 dBm and the receiver sensitivity is configured to be −120 dBm. Isotropic antennas are used at both the BS and the MS at runtime. After that, any required transmit and/or receive antenna pattern and geometry are applied during post-processing, as a spatial-polarisation-phase convolution process. Table 1 shows the parameters that are produced for each ray.

**Figure 2:**
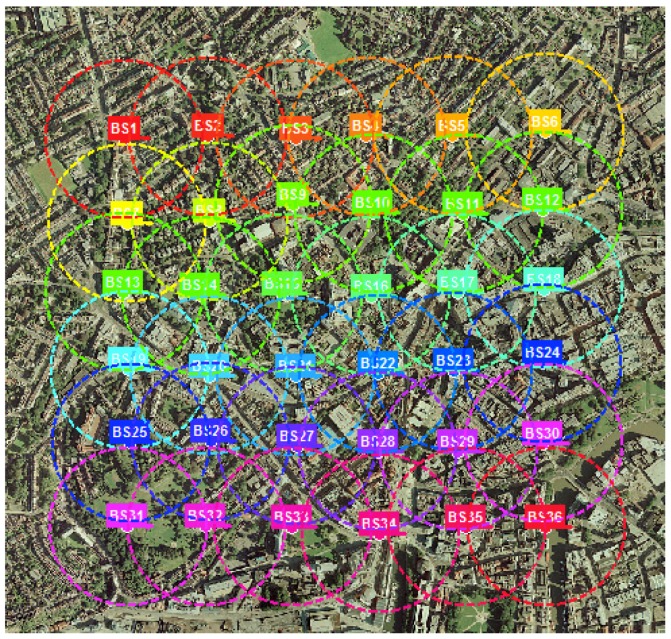
Base station deployment showing the coverage radius.

Hundreds of base station (BS) to mobile station (MS) links were simulated to generate 3D ray data for different areas of the greater city of Bristol. A 6 × 6 BS grid that covers approximately 1 km^2^, is placed on parts of the city. The BS to BS distance is approximately 300 m. The BS deployment is made very dense, mainly to make sure that every position within the area of concern, is covered, thus increasing the likelihood that every MS position will generate enough multipath components between itself and at least a couple of BSs. The deployment also aligns with the idea of densification for boosting capacity in urban centers. The BS deployment for the city center is shown in Figure 2, below.

Because of the random placement of the MSs, some were situated in places that could not receive any useful signal, like in court yards. This meant that, no ray data was generated for those links and consequently those mobile positions were excluded from the study. In all ray-tracing data generation, the parameters are chosen to match those of the massive MIMO testbed at Bristol University [12]. Key outputs from the ray-tracer, that are used in this study, are the BS and MS locations (x and y coordinates), the azimuth AOA at BS, the received power and the time delay, for each ray or path.

### 2.2. Assumptions

- Channel reciprocity

The ray-tracing software is designed to have the BSs acting as signal sources and the MSs as receivers, so channel reciprocity is assumed for any purpose that require uplink transmission.

- Network deployment and capability

Network deployments can be massively different depending on usage. This study assumes a network with base stations that are capable of obtaining reliable AOA information, possibly through the use of antenna arrays, as is the case with massive MIMO Also, the network is assumed to be capable of resolving individual rays or multipath components. Next-generation wireless systems will utilize greater bandwidth than current generation systems, with GHz bandwidths possible at millimeter wave frequencies. Furthermore, bandwidth is not the limiting factor in time resolution as the Cramer-Rao bound on the maximum likelihood timing estimator [13] indicates that SNR is the limiting factor, and so super resolution algorithms can be used to improve timing resolution.

- Noise

Noise in the measured values as presented in the ray-tracer outputs, is neglected. Received power, AOA/AOD information and time delay estimation done in the ray tracing software is considered to be accurate enough for purposes of this study. No noise modelling is built into the algorithms used.

### 2.3. Localisation Algorithms Used for Comparison

The two localization algorithms used to compare the performance of the proposed direct method for localization in LOS and NLOS environments, are TDOA and a hybrid TOA/AOA scheme. TDOA uses 3 Base stations, and one should be the same BS that is used by the other schemes which only require one BS. Detailed information on these algorithms, is provided in [2].

### 2.4. Data Pre-Processing

**Figure 3:**
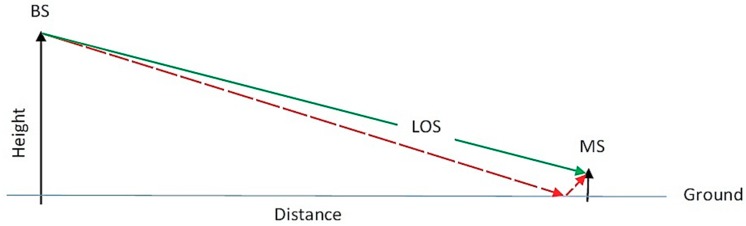
Ground reflected multipath (red dashed path).

The localisation algorithms used in the comparison to the Direct method, made use of the received power, time delay and NLOS/LOS classification, for each path. For each BS-MS link, the first arriving ray or path, is the one whose parameters are used. The assumption is that the first arriving ray represents a LOS path (or a ground reflected path in some cases). This increases the chance of having a data set with LOS rays. On selecting the multipath components (MPCs) for localization, a prioritization approach is adopted, where LOS paths are chosen. If an MS sees multiple LOS paths to multiple BSs (case for TDOA), then those with the shortest delay are selected. If, for example, in TDOA, which requires 3 BSs, an MS has not enough LOS paths, the ground reflected rays are used. Our empirical observations indicated that choosing rays with least time delay produced better localization performance than selecting the rays based on received power level. For the 3 BS TDOA algorithms, each MS selects 3 BSs within its proximity with the least time delays and use those rays for localization. Ground reflected rays are given preference over other NLOS paths because usually ground reflected paths interfere with direct LOS paths. This means that the ground reflected multipath component may be irresolvable from the LOS path since they generally exceed the temporal and spatial resolution capabilities of most measurement systems. The severity of this issue depends on the antenna patterns, the BS/MS heights and how far the mobile station is from the base station [14]. Range error produced by ground reflected (also rooftop diffracted) paths may be smaller than other NLOS scenarios, in most cases. Figure 3, below, demonstrates why ground reflected rays may have time delays comparable to LOS rays.

From the ray-tracing data, ground reflections or rooftop diffractions are determined by rays that exhibit a LOS matching azimuth AOA and AOD but different elevation angles. The total data sets that are selected to be used in these localization algorithms are the same data that was the subjected to the proposed Direct method.

### 2.5. Least-Square Support Vector Machines

Least-Squares Support Vector Machine (LSSVM) is a reformulation of the standard SVMs in order to solve linear kernel-based systems. They were first proposed by Suykens and Vendewalle [15]. They are typically used for classification and regression as is the case in [2,16]. The regression capability is exploited in many applications that require function estimation. In such applications, one moves from a large data set of observations, and tries to construct a model from the data, which is generally counter-intuitive to common engineering approaches where one builds a model and then apply it to new data. The function estimation methodology seeks to construct a function ${ℜ}^{n}\to ℜ$ of the form:

(1) $$y(x)=\sum_{i=N}^{N}\alpha_{i}\psi (x,x_{i})+b,$$

given a training set of *N* data points $\{x_{i}{y_{i}\}}_{i=1}^{N}$ where $x_{i}\in {ℜ}^{n}$ is the $i^{th}$ input and $y_{i}\in ℜ$ is the corresponding “output” to be used for training the regressor. $\alpha_{i}$ are positive real constants and b is a real constant, and both these 2 constants form the parameters of the regressor. The function $\psi (x,x_{i})$ is called the Kernel and it is typically taken to be $x_{i}^{T}x$ for linear SVMs, or $\psi \left(x,x_{i}\right)\;=\;\exp\left\{-\frac{\|x-x_{i}\|{}^{2}}{\sigma^{2}}\right\}$ for Radial Basis Function (RBF) SVMs, with σ, being a constant. The LS-SVM formulation leads to a linear system that can be written in matrix form as:

(2) $$\left[\begin{array}{cc} 0 & 1_{N}^{T}\\ 1_{N} & \Omega +\gamma^{-1}I_{N} \end{array}\right]\left[\begin{array}{c} b\\ \alpha \end{array}\right]=\left[\begin{array}{c} 0\\ Y \end{array}\right],$$

where $Y={\left[y_{1},\;\ldots ,y_{N}\right]}^{T}$, $\alpha ={\left[\alpha_{1},\;\ldots ,\alpha_{N}\right]}^{T}$ and $I_{N}={\left[1,\;\ldots ,1\right]}^{T}$, $I_{N}$ is an N×N identity matrix and Ω is the kernel matrix. The parameter γ tunes the trade-off between model complexity and level of tolerable training errors. The parameters of the regressor α and b are obtained by solving the above linear system (Equation (2)). These parameters are then fed into the regressor (Equation (1)). In this study we choose the Radial Basis Function (RBF) kernel because it gives the best validation and test set performance [17]. The LSSVM formulation and detailed options are available from Vapnik’s original formulation [18].

### 2.6. Direct LSSVM Localisation Method

#### 2.6.1. Training and Estimation

Ray-tracing produces multipath components (MPCs) for each BS–MS link or pair. For each MS position, all the BSs that can be seen by that MS, will produce the MPCs. The BS positions are known. The inputs of the regressor form a (*N* x *5*) matrix whose five columns are: the BS x-coordinate, BS y-coordinate, the signal/ray’s AOA at BS, logarithm of its time delay, and logarithm of its received power. The output sequences used for training, form a column vector with the x-coordinates or the y-coordinates of the MS depending on the coordinates being estimated at that point. Data-points (*N*) in this case therefore, refers to the total number of MPCs that are used, each having the above parameters.

Training data points are created by randomly placing MSs within the coverage area of interest. For each BS-MS link, the first arriving paths are chosen. From these, those that are determined to be LOS are grouped separately to those determined to be NLOS. We determined in [2] that a training data size of at least 3000 data points was sufficient to produce high performing tuning parameters, but in this study, we use data points *N* = 10,000. These datapoints consist of half LOS and half NLOS MPCs. Training data was generated per each considered area and it is that training dataset, that is used for the LSSVM location estimation within that area.

**Figure 4:**
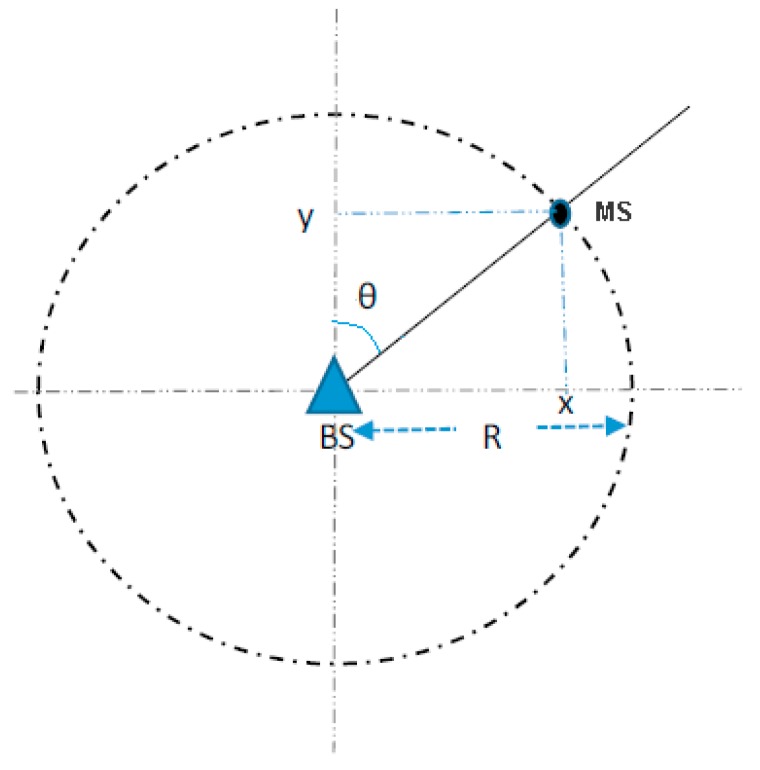
Obtaining the second coordinate for LOS scenarios [3].

Training yields the regressor tuning parameters and constants, which are then used to estimate the coordinates of the MS for any new given data set. Training is done separately for the x and y coordinates using the appropriate output sequences. This approach means estimation of the MS position is O(2) as compared to the traditional regression for NLOS mitigation. It is however possible to just estimate, say, the y-coordinate and use it together with LOS information where available (via NLOS identification or otherwise), to calculate the x-coordinates for those positions that are determined to be in LOS as shown in Figure 4 below.

Once the estimate of the y-coordinate is obtained, the x-coordinate can then be calculated as follows:

(3) $$x_{i}=\;y_{i}\cdot tan\left(\theta_{i}\right),$$

where $x_{i}$ is the x-coordinate corresponding to the y-coordinate $y_{i}$, and $\theta_{i}$ is the AOA. This approach is only suitable for LOS positions. After obtaining the estimates for both the x and y coordinates of the MS, the location error is calculated as in Equation (5). The methodology can be extended to 3D by incorporating the estimation of the elevation coordinate, *z*, in a similar way. As illustrated in Section 2.1, the ray-tracer output data includes the elevation, or height of the MS. This data can be used to training and estimation. Training will have to be done separately for the *z* coordinate and the consequence will be increased computation.

#### 2.6.2. Post-Processing and Outlier Removal

**Figure 5:**
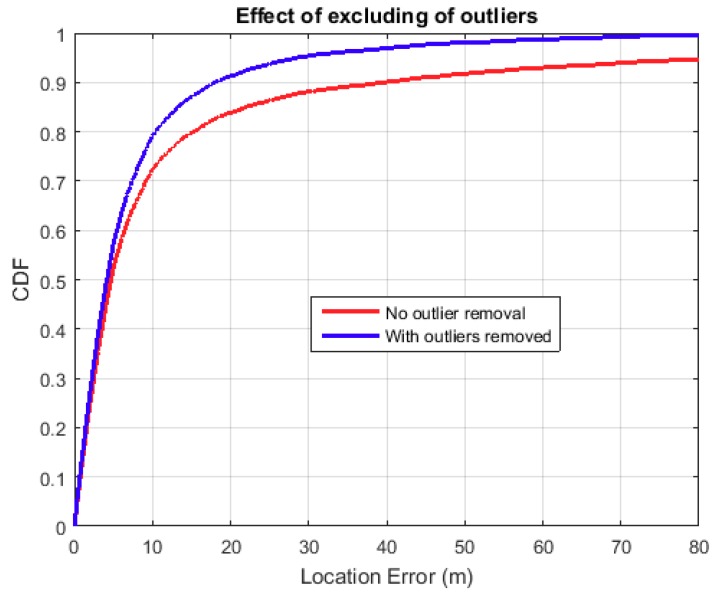
Outlier removal.

The ray-tracing setup has BS-BS distance of 300 m so a coverage radius for each BS of 150 m is considered for determining outliers. The BS deployment seeks to approximate envisaged fifth generation (5G) system deployments, where a dense deployment of small cells is expected. The process of determining and excluding outliers involves calculating the distance $d_{i}$ between the known BS position and the estimated MS location, as follows:

(4) $$d_{i}=\sqrt{\left[{\left(BSx_{i}-\;{\hat{MSx}}_{i}\right)}^{2}+\;{\left(BSy_{i}-\;{\hat{MSy}}_{i}\right)}^{2}\right]},$$

where $BSx_{i}$ is the $i^{th}$ BS’s x-coordinate and ${\hat{MSx}}_{i}$ is the estimated $i^{th}$ MS x-coordinate. The other symbols’ meaning follow, for the y-coordinate. It also follows that for multiple MS positions using the same BS, $BSx_{i}$ and $BSy_{i}$ are constant, for all *i*. A BS receives multiple rays from an MS and each ray is used to estimate the MS position. For a single MS position, some rays will estimate the position better than others, so those rays that result in the BS-MS distance greater than 150 m are discarded. Empirical tests show that more regressor errors start increasing for MS positions beyond 100 m from the BS. Outlier removal criteria may be tightened to any distance but that will create more coverage black spots, hence we settled on 150 m. On average, the total number of data points that were excluded because of this criterion were around 10%. Figure 5, below, shows the effect of excluding those rays that are resulting in outlying MS positions.

### 2.7. Localisation Performance

The positioning error estimate is calculated as the distance between the estimated position and the actual position of the mobile station as obtained from the ray-tracer setup:

(5) $$e_{i}=\sqrt{\left[{\left(x_{i}-\;{\hat{x}}_{i}\right)}^{2}+\;{\left(y_{i}-\;{\hat{y}}_{i}\right)}^{2}\right]},$$

where ($x_{i},y_{i}$) are actual coordinates for the $i^{th}$ MS taken from the ray-tracing tool, and (${\hat{x}}_{i},{\hat{y}}_{i}$) are the corresponding LSSVM estimated MS coordinates. Performance for different scenarios, is compared using the location error cumulative distribution functions (CDF) plots.

### 2.8. Environments Considered

**Figure 6:**
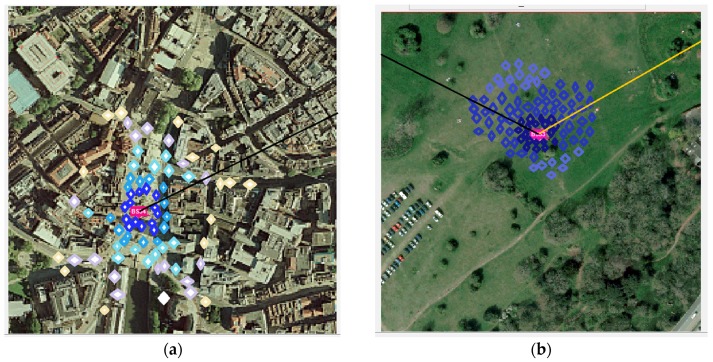
(**a**) Dense urban area/city center (sampled color-coded positions: same color means positions with same received signal power). (**b**) Park/farmland, showing trees and open areas [3].

The three environments considered in [3] are the city center, city peripheral area, and open area (park), but for comparison with the TOA and TDOA localisation schemes, the city center and the park are chosen to represent a dense multipath environment (NLOS), and a LOS scenario, respectively. These are shown in Figure 6 below. Ray tracing is run against each of these areas to generate both the training data, and the data used for performance evaluation.

### 2.9. Performance Comparisons

It is demonstrated in [2] that the TDOA and TOA/AOA localization methods benefit from NLOS mitigation. The process of NLOS mitigation in these cases involve running the LSSVM twice, once for classification and then secondly for regression during mitigation. The direct method discussed herein, also involves running the LSSVM twice, once to obtain the x-coordinate, and second to obtain the y-coordinate. It is therefore of interest to compare the performance of these approaches in different scenarios. It should be noted that the TDOA and TOA/AOA schemes, additionally involve running the localization algorithm itself, after mitigation is applied.

The data for the city environments, was used to run the TOA/AOA algorithm, with NLOS identification and mitigation. By its design, the TOA/AOA algorithm assumes the availability of a LOS component between the BS and the MS, and that, it is the first arriving path, that is LOS. This means that after NLOS identification, using the method described in [2], only those paths that are determined to be LOS are used for localization, meaning that some MS positions could not be localized and were excluded. The LSSVM direct localization scheme was also not run for those positions that were excluded. Because the TOA/AOA scheme in this case is using identified LOS paths, it is the performance of LOS identification and mitigation which is being compared to the direct localization scheme. For that reason, this comparison is only done for one environment, in this case the dense urban environment.

TDOA requires each MS position to be able to see at least three BSs. MS positions that could not see at least 3 BSs were excluded. The LSSVM direct localization scheme uses only one BS and it’s the BS with the shortest time delay to the MS, which is used. To make the localization performance comparison with TDOA, 2 additional BSs are chosen, with best time delays.

## 3. Results

### 3.1. Results for the Direct Method

**Figure 7:**
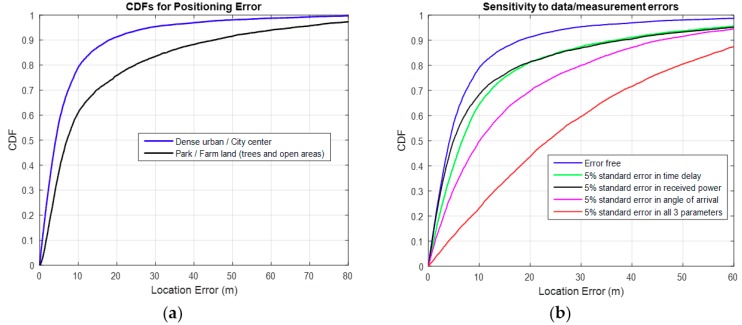
(**a**) Localisation performance for the two environments. (**b**) Sensitivity to measurement errors.

Figure 7a below shows that the Direct localisation approach, performs better in dense urban environments like the city center. This is mainly because it benefits from the uniqueness of the multipath generated in such environments. This can be demonstrated by the fact that, given a set of measurements for received power, time delay and angle of arrival, the probability of getting multiple MS positions that can record similar measurements, from the BS, is small in multipath environments.

Figure 7b shows demonstrates that errors in the data affect the localisation accuracy. These errors could be introduced by electromagnetic noise or equipment errors. For an outage probability of 0.2, errors in all 3 parameters considered, worsens the location accuracy by 40 m. Figure 7b also shows that the Direct scheme is more sensitive to AOA errors than other parameters. AOA error for an MPC in a dense multipath environment can lead to a totally different path between the BS and the MS. An increase in such errors will significantly impact the performance of the scheme. More details on this experiment are contained in [3].

### 3.2. Comparison with TOA/AOA

**Figure 8:**
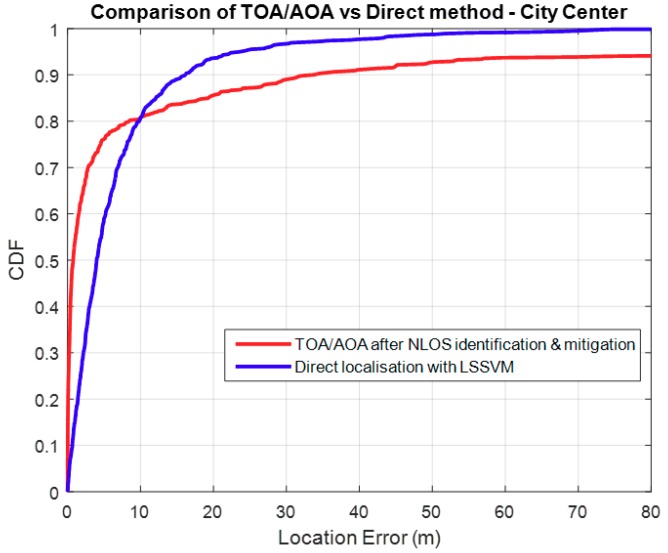
Direct method vs. TOA/AOA.

The comparison of localisation performance between the TOA/AOA and the Direct method is shown in Figure 8, and it shows that the TOA/AOA method performs better if we consider an outage of 20%. The data used was for the urban environment. TOA/AOA performs better because by its design, it uses LOS paths. Where there is no LOS MPCs between the BS and the MS, the algorithm selects ground reflected paths as outlined in the prioritization scheme discussed in Section 2.4. It can be noted that TOA/AOA performance deteriorates if we consider any acceptable outage probability less than 20%. This is mainly due to the percentage of MS positions that do not have LOS paths to the BS. Use of ground reflected paths or any other mitigated NLOS paths results in growing position errors. Identification of LOS paths is covered in [3], together with the subsequent mitigation of NLOS propagation. When the correct AOA is obtained in a LOS link, the source of error then becomes, mainly, the time delay error, which produces the range between the BS and MS. Measurement errors and the positive bias of this delay can be reduced by using the mitigation techniques discussed in [3] and thus the localisation performance can be improved. In a LOS environment TOA/AOA was determined to perform better than the Direct method at any level of acceptable outage.

### 3.3. Comparison with TDOA

**Figure 9:**
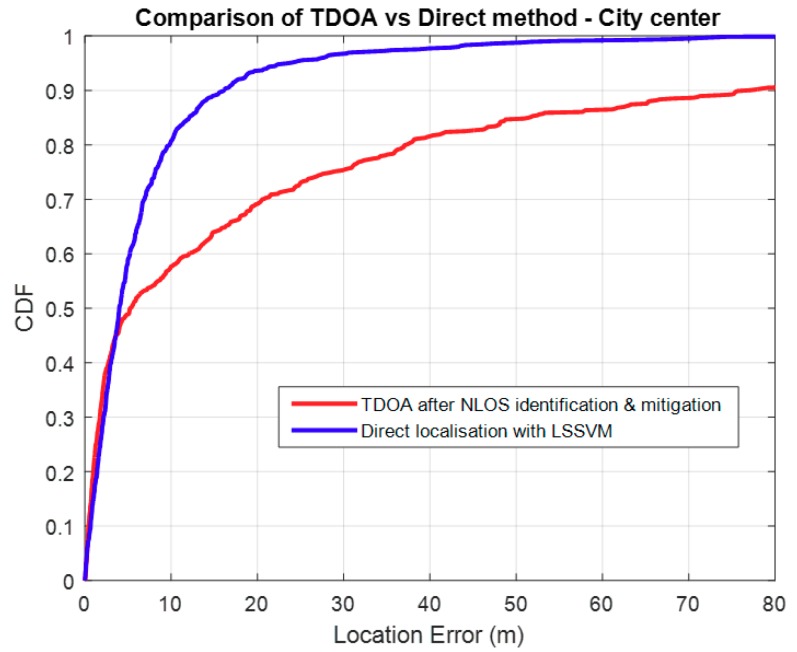
TDOA vs. Direct method in an urban environment.

The LSSVM direct localisation performs better than TDOA in the dense urban environment as shown in Figure 9, below. This is probably because it is benefiting from the uniqueness of multipaths in such an environment. Also, TDOA depends on the performance of NLOS identification and mitigation, so in a case where there are insufficient LOS paths, the time delays used may result in a significantly over-estimated range.

**Figure 10:**
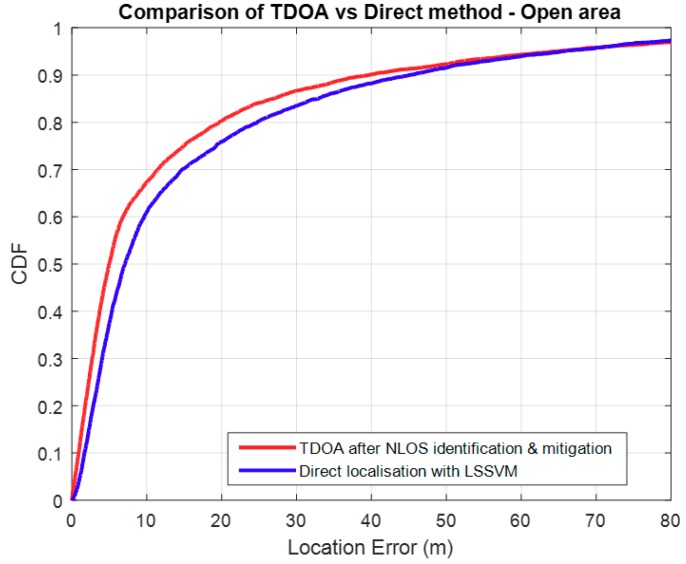
TDOA vs. Direct method in a LOS environment.

Figure 10, below, shows that in open areas, TDOA produces better location accuracy under any given outage probability greater than 0.08. For our chosen standard outage probability of 0.2, TDOA has performs better over the Direct method, by 5m. This is because of the availability and quality of LOS paths. TDOA performance becomes comparable to the Direct method as some NLOS paths start to be included in the algorithm due to unavailability of sufficient LOS paths. Also, some MS positions may suffer from geometric dilution of precision (GDOP) when the 3 BSs chosen are in an undesirable geometry, such as in a straight line. 

## 4. Discussion

The results shown above are location specific. Different performance could be expected with a change in location, but the MS positions considered in this study are in the order of thousands, so it could be expected to produce similar results providing the characteristics of the environment was similar. To that end, an experiment was also conducted using data from a separate area of the city, for the NLOS environment, and a different open area, for the LOS environment, and similar CDF curves were observed.

## 5. Conclusions

This study has demonstrated an approach to urban localisation using ray-traced data and LSSVMs. It demonstrates that the direct localisation approach provides better localisation accuracy compared to the process of NLOS identification and mitigation and then exploiting the traditional localisation algorithms like TDOA and TOA. Because the direct approach is essentially a single BS localisation scheme, it has been demonstrated that AOA errors can greatly affect the location accuracy. In such circumstances, multi BS schemes like TDOA may be able to handle errors in one BS better. Granularity and performance of the Direct method can be further controlled by the size of training data. A larger training data size improves the tuning parameters. Training can also be done per BS, with the tuning parameters stored and referenced per each BS. More BSs can be used to obtain estimates for an MS position, and if each estimate can be assigned some confidence value, the use of multiple BSs can improve accuracy.

The sensitivity of the approach with mobile scatterers is not currently known and requires further study. There are possibilities to mitigate the effects of mobile scatterers by identifying these components through identifying Doppler shift, and eliminating them when comparing against the ray-tracing data. There is an analogy with radar processing in removing clutter, in this case. Further processing using similar techniques, to remove multipath components as a resulting of moving scatterers, will be the subject of further research. Also raising the height of the BSs may provide deterministic multipath components, if the main scatters are assumed to be traffic. Densification through deployment of numerous micro BSs on street lamp posts may actually mean the probability of getting a LOS component increases. Mobility of the MS can also be a function of how fast the snapshot of measurements are processed. Availability of enough processing resources should make periodic estimation possible. Further accuracy for mobility or tracking scenarios can be improved by hybrid data fusion with the MS’s sensor data.

## Figures and Tables

**Table 1:** List of ray-tracer outputs.

Ray-Tracer Output Data
Easting coordinate of BS (*x* coordinate) Northing coordinate of BS (*y* coordinate) Height of BS (*z* coordinate) Easting coordinate of MS Northing coordinate of MS Height of MS Frequency Transmit power Time delay Received power Phase Elevation AOD Azimuth AOD Elevation AOA Azimuth AOA
